# Lysine Acetylation and Deacetylation in Brain Development and Neuropathies

**DOI:** 10.1016/j.gpb.2016.09.002

**Published:** 2017-02-02

**Authors:** Alicia Tapias, Zhao-Qi Wang

**Affiliations:** 1 Leibniz Institute on Aging–Fritz Lipmann Institute (FLI), 07745 Jena, Germany; 2 Faculty of Biology and Pharmacy, Friedrich Schiller University of Jena, 07743 Jena, Germany

**Keywords:** KAT, KDAC, Neural stem cells/neuroprogenitors, Neurogenesis, Neurodevelopmental disorders

## Abstract

Embryonic development is critical for the final functionality and maintenance of the adult brain. Brain development is tightly regulated by intracellular and extracellular signaling. Lysine acetylation and deacetylation are posttranslational modifications that are able to link extracellular signals to intracellular responses. A wealth of evidence indicates that lysine acetylation and deacetylation are critical for brain development and functionality. Indeed, mutations of the enzymes and cofactors responsible for these processes are often associated with neurodevelopmental and psychiatric disorders. Lysine acetylation and deacetylation are involved in all levels of brain development, starting from neuroprogenitor survival and proliferation, cell fate decisions, neuronal maturation, migration, and synaptogenesis, as well as differentiation and maturation of astrocytes and oligodendrocytes, to the establishment of neuronal circuits. Hence, fluctuations in the balance between lysine acetylation and deacetylation contribute to the final shape and performance of the brain. In this review, we summarize the current basic knowledge on the specific roles of lysine acetyltransferase (**KAT**) and lysine deacetylase (**KDAC**) complexes in brain development and the different **neurodevelopmental disorders** that are associated with dysfunctional lysine (de)acetylation machineries.

## Introduction

The brain is the most complex organ in vertebrates and is able to control all other organs in the body. Adaptability and plasticity are the key features of the brain, which grant rapid and coordinated responses to environmental changes. In order to insure the complex function of the brain, a series of mechanisms must work in a coordinated manner starting early on during embryonic development and continuing during postnatal and adult life. Embryonic development defines the size and functionality of the adult brain since the bulk of neurogenesis occurs prenatally while only few neurons are produced after birth [1], [2]. Indeed, the mammalian adult brain has a very limited regenerative capacity following injury compared with other tissues, such as skin, intestine, liver, or lung. This is because of the low amount of neural stem cells (NSCs) in the adult brain and their limited capacity to replace damaged neurons *in vivo* [3], [4], [5]. Therefore, it is essential to tightly regulate the formation, maturation, and maintenance of neurons throughout life to ensure full brain functionality.

At the molecular level, brain development and maintenance are controlled both by cell-intrinsic mechanisms, such as transcriptional programs controlling proliferation and differentiation of NSCs, and extrinsic mechanisms including extracellular cues influencing neuronal migration, synapse formation, and neuronal activity [6], [7]. Epigenetic regulations are important mechanisms controlling brain development, function, and maintenance [8], [9], [10], [11], [12], [13]. Given that epigenetic mechanisms mediate transient or stable heritable changes in the transcriptional programs of cells in response to external and environmental signals, these mechanisms provide an essential pillar for brain adaptability and plasticity [8], [10], [13].

Acetylation of histones is a reversible epigenetic mechanism that can be influenced by external factors [14]. Histone acetylation patterns, which can be directly transmitted to daughter cells, influence other stable chromatin modifications such as histone and DNA methylation, or the recruitment of chromatin modifiers [15]. Increasing evidence has associated dysbalances in histone acetylation with different pathologies affecting the central nervous system (CNS). Moreover, mutations of enzymes and cofactors involved in different types of epigenetic mechanisms are linked to a vast number of neurological disorders [8], [16]. Additionally, lysine or histone deacetylation inhibitors (HDACi) have been proposed as therapeutic approaches for various neurodevelopmental, neurodegenerative, and psychiatric disorders. Several previous review papers have addressed the importance of general or individual epigenetic mechanisms in CNS development, brain function, and maintenance [8], [10], [12], [13], [16], [17], [18], [19], [20], [21], [22]. In this review, we aim to summarize the specific roles of lysine acetylation and deacetylation in brain development. Moreover, we attempt to compile and discuss, for the first time, the different neurodevelopmental disorders associated with mutations or dysfunction of the lysine (de)acetylation machineries.

## Brain development

**Figure 1:**
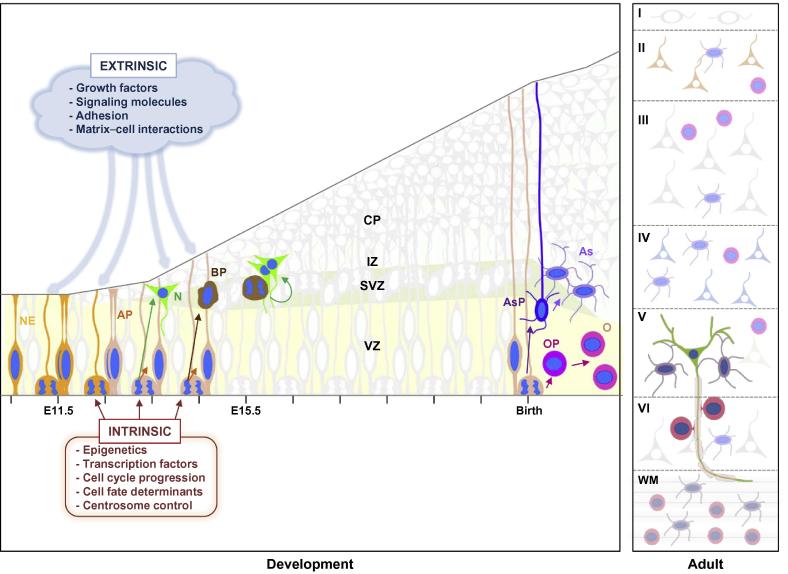
**Mechanisms driving cortical neurogenesis** The scheme illustrates the most important events of neural stem cells during rodent cortical development. Briefly, neuroepithelial (NE) cells are the first stem cells located in the ventricular zone (VZ) and later differentiate into apical progenitors (APs) that give rise to most of the other cell progenies in the cortex. Basal progenitors (BPs) that are located in the subventricular zone (SVZ) mostly divide once to form neurons (Ns). Neurons arising from APs and BPs migrate radially through the intermediate zone (IZ) to the cortical plate (CP), where they reside permanently. After migration, neurons undergo maturation, involving axon growth and synapse formation. Around birth, APs generate astrocyte (As) and oligodendrocyte (O) precursors (AsPs and OPs), which give rise to astrocytes and oligodendrocytes, respectively. Oligodendrocytes mediate axon myelination, whereas astrocytes support mature neurons. The adult cortex is organized in six layers (I–VI) composed of different types of postmitotic neurons, astrocytes, and oligodendrocytes. The cortical white matter (WM) contains the axons of neurons projected to other brain regions, as well as astrocytes and oligodendrocytes. Mechanisms governing brain development can be extrinsic (blue text box) or intrinsic (brown text box). Epigenetics can be influenced by extrinsic mechanisms to modify intracellular programs.

The mammalian CNS arises from the ectoderm germ layer of the early embryo after a series of complex morphological changes. During early development, the neural tube is formed to subsequently generate the early CNS and peripheral nervous system [23]. Brain development begins with the closure of the neural tube and the formation of the forebrain vesicle that contains the neuroepithelium. Neuroepithelial cells are the NSCs and are located along the ventricle in a germinal zone called ventricular zone (VZ). After an early phase of expansion, neuroepithelial cells differentiate into radial glial cells or apical progenitors (APs) that will further generate most of the other cell types in the brain, including different kinds of progenitors, neurons, and glial cells [1] (Figure 1).

The paradigms on how the brain is formed are mainly based on studies conducted on the cerebral cortex, hippocampus, or cerebellum. With the onset of neurogenesis, starting around embryonic day 10.5 (E10.5), the proliferative area within the cerebral cortex is subdivided into the VZ, which contains APs, and the subventricular zone (SVZ), which contains intermediate or basal progenitors (BPs) [1], [24]. Above the proliferative layers, the intermediate zone (IZ) and the cortical plate (CP) contain postmitotic neurons arising from apical and basal neuroprogenitors (NPs) (Figure 1). APs are radially oriented, able to self-renew, and responsible for the formation of neurons. In contrast, BPs mainly divide to produce neurons and are the principal source of cortical neurons (Figure 1). Neurons arising from cortical NPs undergo a process of maturation that starts with their radial migration away from the proliferative area towards their final destination in the CP where they assemble in an inside-out manner, according to the time they arise, generating a distinctive 6-layer structure (Figure 1). Shortly after neuronal differentiation and migration, axons and dendrites connect to form synaptic circuits. During perinatal development, the NP fate shifts to give rise to different types of glial cells, including astrocytes and oligodendrocytes, which provide the essential environment to modulate the chemical and electric signal transduction of neurons [25], [26]. Subsequently, additional steps, including myelin formation, work in a concerted action to shape the final brain cytoarchitecture [27], [28] (Figure 1). To ensure proper function of the brain, all these processes must be tightly controlled.

Indeed, to achieve a fully functional brain, it is essential to control gene expression profiles for the generation of appropriate cell fates. The production of neurons and glial cells, as well as the maintenance of reduced stem cell populations in the adult brain, is accomplished by the complex interplay of intrinsic and extrinsic signals. Therefore, cell autonomous mechanisms including intrinsic transcriptional programs that control cell cycle progression, centrosome activity, or the polar distribution of cell fate determinants, are of great importance [29], [30], [31]. Equally important, extrinsic cues, including growth factors, cytokines, adhesion molecules, and extracellular matrix components, also play essential roles during brain development [27], [32], [33], [34], [35]. Remarkably, epigenetic mechanisms, which can integrate extracellular with intracellular signaling and can be inherited through cell divisions, are emerging as prominent mechanisms for the determination of the final shape of the adult CNS [12], [36]. Epigenetic mechanisms have the ability to regulate gene transcription and other DNA-dependent cellular processes by altering chromatin structure without affecting the DNA sequence. These mechanisms include, among others, chemical modifications of the DNA, such as DNA methylation or hydroxymethylation, histone variant exchange, non-coding RNAs, changes in nucleosome positioning, as well as posttranslational modifications of histones, such as acetylation, methylation, phosphorylation, poly ADP-ribosylation (PARylation), and ubiquitination [37], [38], [39].

## Lysine acetylation and deacetylation

**Figure 2:**
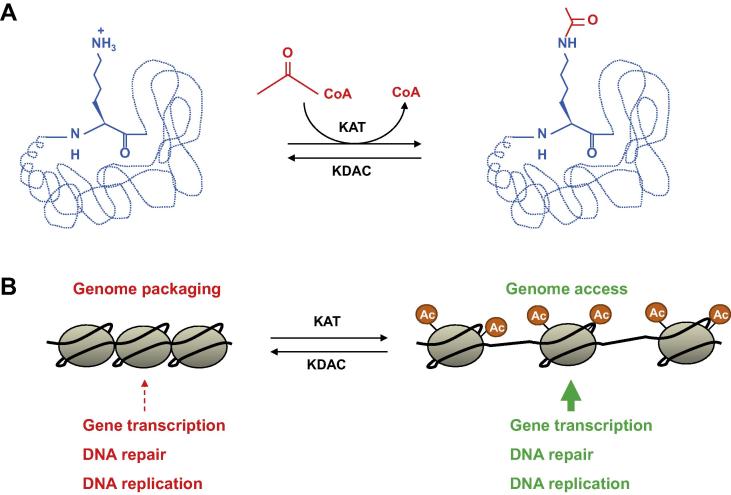
**Lysine (de)acetylation and its function in chromatin remodeling** **A.** Lysine acetylation involves the transfer of the acetyl group from acetyl-CoA (red) into a target protein (blue) mediated by KATs. The reversible reaction is mediated by KDACs. **B.** Simplified scheme showing the effects of histone acetylation on the chromatin structure and the resulting consequence for DNA-dependent processes. Gray circles represent nucleosomes and the black lines represent DNA. KAT, lysine acetyltransferase; KDAC, lysine deacetylase.

Lysine acetylation and deacetylation represent prominent posttranslational modifications of histone tails that influence chromatin structure and epigenetic states. Lysine acetylation is mediated by lysine or histone acetyltransferases (KATs or HATs), whereas lysine deacetylation is catalyzed by lysine or histone deacetylases (KDACs or HDACs) [40], [41], [42], [43] (Figure 2A). Generally, histone acetylation correlates with higher transcriptional activity owing to a higher accessibility of the transcriptional machineries to DNA, whereas histone deacetylation is correlated with a lower transcriptional activity owing to chromatin condensation [38] (Figure 2B). There are different families of KATs and KDACs, which present both specific as well as overlapping functions. KATs and KDACs are mainly found as a part of multiprotein complexes, and their activity and substrate specificity depend on the composition of the whole complex rather than on single enzymes. Most of the enzyme complexes are found both in the nucleus and cytoplasm, indicating multiple functions in (de)acetylation of histones and other proteins. However, some enzyme complexes are specifically either nuclear or cytoplasmic, indicating more specific functions [40], [41], [42] (Table 1 and Table 2). KATs and KDACs need to work in a concerted action in order to regulate transcriptional profiles and other cellular processes, such as microtubule dynamics, by maintaining the appropriate level of lysine acetylation at histone tails and other proteins, such as tubulin [44].

KATs, which are evolutionarily conserved from yeast to humans, catalyze the transfer of acetyl groups from acetyl-CoA onto lysine residues of acceptor proteins (Figure 2). There are three major KAT families based on sequence similarities (Table 1). The general control of amino acid synthesis protein 5-like 2 (GCN5)-related *N*-acetyltransferases (GNATs) include different families of KATs that share similar structural features and functional roles. The GNAT superfamily in humans includes HAT1 (KAT1), GCN5 (KAT2A), p300/CREB-binding protein (CBP)-associated protein (PCAF; KAT2B), elongator acetyltransferase complex subunit 3 (ELP3; KAT9), cysteine rich protein 2 binding protein (CSRP2BP; KAT14), activating transcription factor 2 (ATF-2), and HAT4 [22], [45]. p300/CBP family is composed of two closely-related members, CBP (KAT3A) and p300 (KAT3B). They are among the most studied KAT enzymes, especially in relation to histone acetylation and transcriptional regulation [46]. The MYST (MOZ, Ybf2, Sas2, and TIP60) family is composed of five members in humans, known as Tat-interacting 60 kDa protein (TIP60; KAT5), monocytic leukemia zinc finger related protein (MOZ; KAT6A), MOZ-related factor (MORF; KAT6B), HAT bound to origin recognition complex 1 (ORC1) (HBO1; KAT7), and human males-absent on the first (MOF; KAT8). Additionally, a group of other proteins exhibit KAT activity but lack common structures. These include TATA box binding protein (TBTP)-associated factor, 250 kDa (TAF1; KAT4), transcription factor IIIC subunit delta (TFIIIC; KAT12), nuclear receptor coactivator 1 (NCOA1; KAT13A), NCOA3 (KAT13B), NCOA2 (KAT13C), and circadian locomotor output cycles kaput protein (CLOCK; KAT13D).

KDACs are enzymes responsible for the removal of acetyl groups from lysine residues (Figure 2). In contrast to KAT enzymes, which are mostly ubiquitous, KDACs present specific expression patterns that determine their function within different cell types (Table 2). KDAC proteins are divided into four classes based on functional and sequence similarities (Table 2). The activity of classes I, II, and IV depends on zinc, whereas the activity of Class III KDACs depends on NAD^+^ [19], [47]. Class I contains the ubiquitous enzymes HDAC1, HDAC2, HDAC3, and HDAC8, which have been extensively studied in the context of histone deacetylation. Except for HDAC8, all Class I enzymes are part of prominent transcriptional corepressor complexes such as Sin3, Mi2/nucleosome remodeling deacetylase (NuRD), repressor element 1-silencing transcription factor (REST) corepressor 1 (CoREST), or nuclear receptor co-repressor 2 (N-CoR)/silencing mediator for retinoid or thyroid-hormone receptors (SMRT), thereby mediating transcriptional repression [48]. Class II consists of tissue-specific enzymes, which are further divided into Class IIA, including HDAC4, HDAC5, HDAC7, and HDAC9, and Class IIB, including HDAC6 and HDAC10. Class III contains sirtuins SIRT1–3 and SIRT5–7, which are able to sense NAD^+^ levels and play important roles in the regulation of cellular homeostasis, metabolism, and lifespan [49]. Class IV, one of the less-studied KDAC groups, contains HDAC11 only, owing to the lack of homology with other KDACs [50].

Contrary to what was initially thought, lysine acetylation also occurs in a vast number of proteins besides histones. Protein acetylation has emerged as an important posttranslational modification regulating many cellular processes [51]. Hence, the balance between the activity of different KATs and KDACs not only influences cellular functions through epigenetic transcriptional regulation but also directly modulates multiple signaling pathways. Remarkably, the activity of both KATs and KDACs is closely related to metabolism and nutrient availability. Indeed, all KAT enzymes directly depend on acetyl-CoA levels. For instance, sirtuins require NAD^+^, whereas Class I, II, and IV KDACs need zinc in order to function [14]. Therefore, lysine acetylation represents a key step to integrate environmental signals, such as exercise, diet, maternal care, or xenobiotic exposure, for the regulation of cellular responses involved in adaptive processes [51], [52], [53], [54]. Consequently, it is reasonable to hypothesize that lysine acetylation might highly influence the final brain shape through multiple mechanisms in response to the cellular context.

## Lysine acetylation and brain development

Abnormal lysine acetylation activity or histone acetylation levels have been linked to different pathological conditions or alterations in brain development both in humans and in mouse models (Table 3). Mutations in some KATs or KDACs have been identified in various human developmental diseases (Table 3). Furthermore, mouse models carrying mutations in KAT and KDAC enzymes and components have corroborated the involvement of these enzymes in brain development (Table 3).

### Functions of KATs during brain development

Numerous studies using cellular and animal models have been performed to unravel the function of different KAT and KDAC complexes during brain development. Generally, these studies have focused on the effects of histone acetylation on important developmental transcriptional programs. Moreover, the acetylation of proteins other than histones has also been related to different neurodevelopmental processes such as NP fate determination and neuronal migration [55], [56]. Most of the KAT complexes are essential during developmental stages as evidenced by early embryonic lethality in mice devoid of KATs. The deletion of KAT enzymes in mice often dramatically affects brain development. However, given the substrate specificity of each single KAT complex, the disruption of different complexes leads to different outcomes (Table 3). Hence, there are only a few studies that have addressed the exact roles of specific KAT enzymes and complexes in the CNS development.

#### p300/CBP KATs

p300/CBP family members are required for proper brain development and mutations in the genes encoding these proteins are associated with the human Rubinstein-Taybi syndrome (RSTS) [57]. Although sharing 86% amino acid sequence homology [58], p300 and CBP have both overlapping and distinct functions. Both *p300* and *Cbp* null mouse embryos, as well as double heterozygous *Cbp/p300* embryos, exhibit defects in neural tube closure and die before E12 [59]. In contrast, adult single *p300*-knockout or *Cbp*-knockout heterozygous mice are viable, and have the normal general brain morphology and long-term memory despite exhibiting impaired behavior at birth [60], [61], [62]. Moreover, decreased CBP activity by heterozygous *Cbp* knockout or *Cbp* siRNA knockdown results in decreased differentiation of cortical NPs and neural precursors into neurons and glial cells within the ganglionic eminence, without affecting their survival or proliferation [61], [63]. Chromatin immunoprecipitation (ChIP) analyses of wild type cortices reveal that CBP is associated with promoters of neural differentiation genes (such as *α1-tubulin*), and glial differentiation genes (such as *Mbp* encoding myelin basic protein and *Gfap* encoding glial fibrillary acidic protein). These analyses are suggestive of an involvement of CBP in the transcriptional activation of NP differentiation genes [61]. Strikingly, treatment with trichostatin A (TSA), an inhibitor of Class I and II KDACs, completely rescues the differentiation defects of NPs demonstrating that CBP KAT activity is necessary for NP differentiation [61], [63]. Similarly, Zhang et al. [56] have recently demonstrated that p300, in cooperation with HDAC3, promotes oligodendrocyte differentiation involving not only the acetylation of histones but also the direct acetylation of the transcription factor signal transducer and activator of transcription 3 (STAT3). Altogether, these studies show a critical role of p300/CBP family members in the regulation of NP differentiation, and hence brain development.

#### GNAT KATs

GCN5 and PCAF show 89.5% amino acid sequence homology, whereas other GNAT family members are more diversified [22]. Complete knockout of *Pcaf* does not seem to show noticeable effects in mice during embryonic development [64]. Interestingly, deletion of *Gcn5* leads to embryonic lethality in mice around E10.5. These mice exhibit severe developmental defects, such as growth retardation, increased apoptosis, as well as failure to generate the neural tube and dorsal mesoderm-derived lineages [65]. Moreover, double knockout of *Pcaf* and *Gcn5* leads to more severe defects and lethality around E7 [65]. These findings suggest a unique role for GCN5 and an overlapping role with PCAF. Further studies demonstrate that mice bearing different *Gcn5* mutations, including a hypomorphic *Gcn5* mutation, a catalytically-dead *Gcn5* point mutation, and a double knockout *p53*/*Gcn5* mutation, exhibit exencephaly and neural tube closure defects and die pre- or peri-natally [66], [67], suggesting that both KAT activity and protein expression of GCN5 are essential for proper embryo and brain development. Interestingly, tissue-specific deletion of *Gcn5* in the CNS by *Nestin*-Cre expression results in microcephaly and a decrease in the NSC mass due to decreased cell proliferation that is associated with lower expression of N-Myc target genes [68]. Moreover, despite normal embryonic development, adult *Pcaf* knockout mice exhibit an impaired short-term memory two months after birth, accompanied by subtle differences in hippocampal morphology. Progressively, these mice exhibit an age-related decline in short- and long-term memories [69], [70]. Additional studies on KAT elongator complex during brain development have reported that knockdown of both *Elp1* and *Elp3* causes decreased neuronal migration and reduced levels of α-tubulin acetylation, thus identifying a key role of these molecules in regulating the migration and maturation of projection neurons [55]. Therefore, different GNAT family members play unique roles to control different steps of the neurogenic process, including NP proliferation, fate determination, neuronal maturation, and migration.

#### MYST KATs

Although mutations in the MYST family members often lead to neurodevelopmental defects in humans, not many studies have been conducted to address their specific roles during brain development. This may be due to the fact that while heterozygous mutations of these factors exhibit no phenotypes, homozygous mutations of most of these members, including TIP60, HBO1, and MOF, as well as cofactors like transformation/transcription domain-associated protein (TRRAP) or DNA methyltransferase 1 associated protein 1 (DMAP1), lead to early embryonic lethality around the blastocyst stage or post gastrulation [71], [72], [73], [74], [75]. These observations show an essential role of these complexes in early embryonic development and also indicate technical difficulties to conduct neurodevelopmental studies.

*Morf* was first identified in a mouse gene-trap screen for genes important in neural development [76]. MORF is highly expressed in the neurogenic regions of the developing and adult brain [76], [77]. Interestingly, although mutations of *MORF* cause different neurodevelopmental disorders in humans, the mechanisms through which MORF regulates brain development are not fully understood [78], [79]. Notably, knockout mice of *Moz*, coding for a closely-related KAT with MORF, have been generated by two different laboratories. These mice exhibit embryonic or perinatal lethality, craniofacial abnormalities, and defects in the hematopoietic system [80], [81]. However, there are no data available on their brain morphology. Interestingly, cellular studies have shown that MOZ KAT activity is required for the repression of p16^INK4a^ in NSCs in order to avoid replicative senescence [82], suggesting a role of MOZ in regulating NP maintenance. Similarly, the transcriptional activity of PAX6, the master regulator of neurogenesis, has been shown to be enhanced by TIP60 during retinal development, suggesting a role of TIP60 during neurogenesis [83].

TRRAP is an essential cofactor of different GNAT and MYST family KAT complexes [84]. In agreement with other knockout mouse models of MYST family members, complete knockout of *Trrap* leads to early embryonic lethality around the blastocyst stage [74]. Additionally, specific deletion of *Trrap* in the embryonic brain by using *Nestin*-Cre causes cell proliferation defects accompanied by premature AP differentiation, leading to severe brain atrophy. Strikingly, unscheduled differentiation of *Trrap*-deleted NPs is attributed to cell cycle defects [85]. Similarly, forebrain-specific deletion of the gene encoding the bromodomain and PHD finger-containing protein 1 (BRPF1), a common cofactor of MOZ, MORF, and HBO1 KAT complexes, leads to dentate gyrus hypoplasia and reduced expression of key genes in NP maintenance during hippocampus development [86]. Altogether, these studies show that the MYST family members are essential for brain development.

### Functions of KDACs during brain development

The balance between lysine acetylation and deacetylation is as much dependent on KATs as it is on KDACs. In fact, it is the kinetics of KAT versus KDAC activity that dictates the final acetylation status of histones and other proteins, thus determining subsequently their functionality [44]. Indeed, a bulk of evidence suggests that KDACs, like KATs, play very important roles during brain development (Table 3).

#### Class I KDACs

Although sharing 86% amino acid sequence homology and binding to the same transcription repression complexes, HDAC1 and HDAC2 display different expression patterns during brain development, thus indicating their specific functions in this process. Strikingly, *Hdac1* is expressed in NPs, whereas *Hdac2* is expressed in post-mitotic neurons in the CNS of mice at E13.5 [87]. Knockout of *Hdac1* or *Hdac2* alone causes lethality, although at different developmental stages [88]. Moreover, while specific ablation of *Hdac1* or *Hdac2* in the mouse CNS using *Nestin*-Cre does not have any obvious consequences on brain development, combined deletion of *Hdac1* and *Hdac2* results in embryonic or perinatal lethality, due to cell death and failure of NPs to differentiate into mature neurons [88], [89]. Interestingly, *Hdac1*^+/-^*Hdac2*^-/-^ mice display impaired brain development and perinatal lethality, due to reduced NP proliferation and premature differentiation mediated by overexpression of protein kinase C γ (PKCγ) [89]. Altogether, these studies suggest that HDAC2 plays a unique and important role in controlling the fate of NPs during brain development.

The deletion of *Hdac3* in the CNS using *Nestin*-Cre causes perinatal lethality, which is accompanied by major abnormalities in the cytoarchitecture of the neocortex and cerebellum [90]. Specifically, these mice show an increase in the number of astrocytes concomitant with a decrease in the number of oligodendrocytes and a mislocalization of neurons in the cortex and cerebellum. These findings suggest a role of HDAC3 in cell fate determination of NPs and in neuronal migration [90]. Zhang et al. [56] have recently reported that HDAC3, in cooperation with p300, is important for the regulation of the oligodendrocyte-astrocyte differentiation switch through acetylation of histones and STAT3. Similarly, it has been reported that acetylation of the oligodendrocyte transcription factor OLIG1 drives its translocation from the nucleus to the cytoplasm, thereby regulating oligodendrocyte maturation. The (de)acetylation of OLIG1 is mainly mediated by CBP and HDAC1 [91]. Altogether, these studies indicate that Class I KDACs are important for the regulation of multiple steps during brain development. It is worth noting that *Hdac8* knockout mice exhibit perinatal lethality due to massive ossification defects in the skull, which lead to brain tissue herniation and brain hemorrhage, suggesting a specific role of HDAC8 in regulating skull morphogenesis rather than brain development [92].

#### Class II and Class IV KDACs

Class II KDACs exhibit specific expression patterns during embryonic development and adulthood. Nonetheless, their roles during brain development remain mostly unknown. Many studies have linked HDAC4 with neuronal survival in cell cultures [93]. Intriguingly, *Hdac4*-null mice die perinatally due to severe bone malformations and chondrocyte hypertrophy [94]. Moreover, conditional deletion of *Hdac4* in the brain using *Thy1-* or *Nestin*-Cre does not result in any obvious defects. These *in vivo* studies suggest that HDAC4 does not play important roles during brain development or its function is compensated for by other KDACs [95]. Similarly, *Hdac9* and *Hdac6* knockout mice show no obvious neural phenotypes [96], [97]. Interestingly, Liu et al. [98] have shown that HDAC11, a Class IV KDAC, is expressed soon during postnatal development in mature neurons and oligodendrocytes. Expression of HDAC11 correlates with a decrease in histone acetylation, implying a specific role of HDAC11 in the development or maturation of oligodendrocytes and neurons.

#### Class III KDACs

Among the sirtuin family members, SIRT1 and SIRT2 seem to play opposite roles during brain development. Different studies have been performed using cellular systems, *in utero* electroporation, and knockout models to investigate their functions. It has been shown that SIRT1 regulates the cell fate of NPs and is required for neurite outgrowth, axonogenesis, and dendritic branching through multiple mechanisms including the activation of AKT/glycogen synthase kinase 3 (GSK3) signaling and inhibition of pro-neural genes from the Notch-hairy/enhancer of split (Hes) axis [99], [100], [101]. In contrast, deacetylation of tubulin and microtubules by SIRT2 has been shown to inhibit neurite outgrowth and oligodendrocyte differentiation [102], [103]. Altogether, the concerted action of SIRT1 and SIRT2 modulates the differentiation of NPs and the maturation of neurons. Moreover, it has been shown that *Sirt6*-null mice, display profound abnormalities, including low insulin, hypoglycemia, and premature aging, and die within four weeks after birth [104]. Specific deletion of *Sirt6* in the brain using *Nestin*-Cre leads to postnatal growth retardation in mice due to low levels of growth hormone and insulin-like growth factor 1 (IGF-1) through yet unknown mechanisms [105].

#### Effects of chemical HDACi on brain development

There are a remarkable number of studies investigating the effects of HDACi on neurodevelopmental processes *in vivo* and *in vitro* in mice and humans*.* All have shown that inhibition of KDACs causes dramatic effects during brain development. In humans, treatment with valproic acid (VPA), an inhibitor for Class I and II HDACs, during pregnancy is associated with the occurrence of autism in the progeny [106], [107]. Additionally, postnatal treatment of mice with TSA or VPA leads to the dysregulated activity of adult NPs and reduction in NP progenies [108]. Moreover, different cellular studies have shown that treatment of murine NPs with TSA or VPA leads to lower cell proliferation and higher neuronal differentiation, concomitant with a decreased astrocytic differentiation [109], [110], [111]. Indeed, it has been reported that there exists an association between the VPA-induced increase in histone acetylation at pro-neural genes of mouse NPs and an increase in neuronal formation [111]. Interestingly, VPA reduces axonal growth and leads to impaired synapse formation in rat cortical neurons [112], but stimulates the proliferation of rat glial precursors in culture [113]. Moreover, HDACi can expand the differentiation potential of cultured rat oligodendrocyte progenitor cells to generate neuronal lineage through a mechanism that involves the reactivation of NSC genes including *Sox2* [114]. Altogether, these studies show that the modulation of acetylation levels by HDAC inhibitors could lead to tremendous effects on brain development.

## Human disorders caused by abnormalities in KAT or KDAC activity

### Disorders caused by genomic mutations

Given the important role of KATs and KDACs in controlling transcription and other cellular processes, most of the mutations in the genes encoding these enzymes are likely to be incompatible with life. However, owing to a high degree of substrate redundancy and the presence of hypomorphic mutations, a number of human syndromes have been mapped to mutations on both KAT and KDAC coding genes. Common features of these syndromes are brain abnormalities, usually including primary microcephaly, mental retardation, global developmental delay, and craniofacial dimorphism [115]. Interestingly, as detailed below, individuals with mutations in KAT coding genes suffer more from additional multi-organ defects than those presenting mutations in KDAC coding genes.

Mutations in genes encoding CBP/p300 family members lead to RSTS, a congenital autosomal dominant disorder also known as broad thumb-hallux syndrome (OMIM 180849 and 613684) [57]. RSTS is characterized by microcephaly, mental retardation, postnatal growth deficiency, broad thumbs and halluces, and dysmorphic facial features [115]. This disorder was first described in 1963 and had an estimated incidence in 1990 of 1 in 100,000–125,000 live births [116]. Mutations in the *CBP* gene have been reported in approximately half of the RSTS patients [117], [118], whereas mutations in the *p300* gene are estimated to represent 5%–8% of all cases [119], [120], [121], [122], [123], [124]. Remarkably, RSTS patients exhibit neuroanatomical defects, such as agenesis of the corpus callosum and cortical clefts, among others, and a range of neurological phenotypes including poor motor coordination, short attention, autistic features, seizures, and abnormal electroencephalograms [115]. These patients are also prone to brain tumors [115]. Similarly, *CBP* gene duplications cause a disorder called 16p13.3 duplication syndrome (OMIM 613458), which is characterized by a mild to moderate intellectual disability (ID) and abnormal facial and skeletal morphology [125]. The fact that both deletions and duplications of *CBP* cause similar brain manifestations shows that a tight control of CBP level and function is essential for efficient brain development. Moreover, de Vries et al. [126] have recently shown that mosaic *CBP* mutations cause overlapping features of RSTS and Filippi syndrome (OMIM 272440). Filippi syndrome is usually caused by mutations in the mitotic spindle protein cytoskeleton associated protein 2-like (*CKAP2L*) and is characterized by microcephaly, short stature, syndactyly, intellectual disability, and facial dysmorphism (OMIM 272440).

It is of note that, probably due to lethality, to date, no syndromes have been linked to mutations in genes encoding GNAT KAT family members. Analysis of rare variants in neurological disorders identified an association between human neurological diseases and CRP2BP [127]. However, a causal relationship and possible mechanisms have not been addressed.

Different gene mutations leading to C-terminal truncations of MORF cause the rare genitopatellar syndrome (OMIM 606170), a condition characterized by microcephaly, severe psychomotor retardation, ID, genital abnormalities, missing or underdeveloped kneecaps, and other abnormalities [78]. Mutations of *MORF* are also responsible for Say-Barber-Biesecker variant of Ohdo syndrome (OMIM 603736), characterized by severe mental retardation, distinctive facial appearance, and other skeletal problems [79]. Additionally, *MORF* haploinsufficiency has been proposed as a rare cause of Noonan syndrome-like disorder that is characterized by short stature, retarded bone age, attention deficit hyperactivity with learning disability, and distinct facial features [128]. Altogether, these studies highlight a crucial role of the full-length MORF protein during embryonic development and, specifically, during brain development. In addition, heterozygous nonsense mutations in the gene encoding the closely-related MYST member MOZ have been identified as a frequent cause of syndromic developmental delay with microcephaly and dysmorphic mutations. This syndrome is linked with alterations in global acetylation of H3K9 and H3K18 and p53-mediated pathways [129]. Interestingly, *BRD1* that encodes bromodomain-containing protein 1, an essential cofactor of MOZ/MORF/HBO1 complexes, has been repetitively identified as a susceptibility gene for schizophrenia and bipolar disorder [130], [131], [132]. These findings suggest that mutations of MYST KATs may lead to mild brain developmental defects, thereby causing psychiatric disorders.

Mutations in *HDAC8*, which encodes the class I KDAC enzyme, have been identified in Wilson-Turner X-linked mental retardation syndrome (OMIM 309585), a neurological disorder characterized by ID, dysmorphic facial features, hypogonadism, short stature, and truncal obesity [133]. *HDAC8* mutations are also found in Cornelia de Lange-like syndrome, which is characterized by, among other features, distinct facial features, growth failure, and ID [134]. Furthermore, *HDAC4*, which encodes the class IIA KDAC enzyme, is located on the chromosome 2q37 whose heterozygous loss is associated with chromosome 2q37 deletion syndrome, also called brachydactyly-mental retardation syndrome (OMIM 600430). This syndrome shows mild to moderate mental disabilities, behavioral abnormalities, dysmorphic facial features, brachydactyly type E, and short stature [135], [136]. Additionally, a hemizygous deletion of *HDAC9* has been identified in a small proportion of schizophrenia patients [137]. Moreover, mutations in the 3′ untranslated regions (UTR) of *HDAC6*, which encodes the class IIB KDAC enzyme, suppress miR433-mediated posttranscriptional regulation and cause overexpression of *HDAC6*, resulting in chondrodysplasia with platyspondyly, distinctive brachydactyly, hydrocephaly, and microphtalmia syndrome (OMIM 300863), which exhibits, among other symptoms, hydrocephaly and macrocephaly [138], [139]. Taken together, these findings suggest an essential role of KDACs during brain development in humans and argue for distinct functions of different KDACs during this process.

### Disorders influenced by KAT/KDAC activity

Autism spectrum disorder (ASD) includes different disorders characterized by persistent deficits in social communication, interaction, and repetitive patterns of behavior during early childhood with significant functional impairments later in life [107]. Although many ASD risk factors including genetic factors, neuroanatomical abnormalities, and prenatal and perinatal environmental factors have been identified, the specific causes contributing to the development of ASD remain unknown. Lysine acetylation and deacetylation have been previously linked to ASD. Increase in *HDAC9* copy number has been correlated with an increased risk of autism [140], whereas according to the Simons foundation autism research initiative (SFARI) [141] and Autism KB [142] databases for genetic variants associated to ASD risk, different KATs and KDACs are associated with ASD risk. Additionally, mutation in the CH1 domain of CBP results in autism-relevant behaviors in mice [143]. Moreover, *in utero* exposure to VPA correlates with ASD occurrence in humans and rodents [106], [107]. Indeed, VPA is currently used in animal research to model this disease. Strikingly, VPA administration in pregnant rats correlates with an upregulation of PAX6 target genes in the offspring, leading to abnormal neurogenic patterns in the developing brain [144]. Accordingly, since neurogenesis occurs primarily prenatally, the postnatal administration of VPA in animal models does not correlate with ASD, but, interestingly, rather ameliorates ASD symptoms [145], [146].

The Rett syndrome (RTT; OMIM 312750) is an X-linked neurodevelopmental disorder caused by mutations in *MEPC2*, which encodes methyl CpG binding protein 2 that is able to regulate transcription [147]. RTT affects mainly women and is characterized by microcephaly as well as developmental and mental retardation, among other features. Interestingly, various reports have linked disturbances in lysine acetylation with RTT. Analysis of cells derived from RTT patients revealed hyperacetylation of histone H4 [148], whereas mouse models with a truncated form of MECP2 resemble RTT and display hyperacetylated histone H3 [149]. Moreover, MEPC2 itself can be acetylated by p300 and deacetylated by SIRT1 in cultured cells and the function of MECP2 is modulated by such (de)acetylation [150], [151]. Given the crosstalk between MEPC2 and KATs/KDACs, HDAC inhibitors have been proposed as a therapeutic approach to treat RTT [152], [153]. Similarly, histone (de)acetylation seems to play a role in the fragile X-linked syndrome (OMIM 300624), which is the second most common cause of mental impairment after trisomy 21 and characterized by moderate to severe mental retardation, macroorchidism, and distinct facial features [154]. The fragile X-linked syndrome is caused by a CGG-triplet repeat expansion in the 5′ UTR of *FMR1*, resulting in extensive local methylation, silencing of *FMR1*, and loss of fragile X mental retardation protein (FMRP). Given the extensive chromatin rearrangements caused by the CGG-triplet repeat, HDAC inhibitors have been used in different models to reactivate the transcription of *FMR1* [155]. Accordingly, treatment with VPA has been reported to ameliorate hyperactivity in fragile X syndrome boys [156].

Several studies have highlighted the importance of maternal behavior both prenatally and perinatally on lysine acetylation and its consequences on brain development. Guo et al. [157] have described a decrease in CBP levels in the cerebellum of rat models of fetal alcohol spectrum disorders, which are a group of conditions characterized by microcephaly, poor coordination, low intelligence, and behavioral abnormalities caused by maternal consumption of alcohol during pregnancy. Furthermore, a wealth of evidence has suggested that negative environmental stimuli during postnatal development, such as parental misconduct or other stressful situations, have an important impact on the onset of anxiety disorders [158]. Indeed, different studies have shown that maternal care influences hippocampal morphology and function through changes in the epigenome that lead to a modulation of glucocorticoid receptor signaling [159]. Interestingly, these effects can be reversed by HDACi, showing that the plasticity of histone acetylation plays a pivotal role in these processes [159]. Strikingly, various translational studies have observed a decrease in the expression of glucocorticoid receptors in individuals with a history of childhood adversity [160], [161], [162], [163]. Hence, these studies agree with the involvement of the epigenetic regulation of corticoid receptor signaling in response to adversity during childhood in humans [9]. These findings show that lysine acetylation levels can be influenced by extracellular factors and have dramatic effects on brain development and, subsequently, on brain function.

## Concluding remarks and future directions

**Figure 3:**
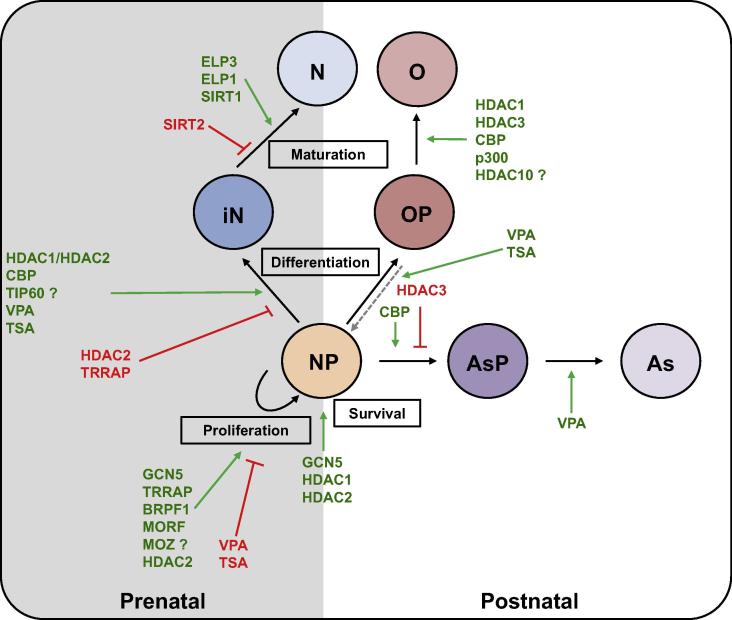
Molecular pathways in neural stem cells and their progenies KATs and KDACs modulate various neurodevelopment processes prenatally (gray background) and postnatally (white background). KATs and KDACs play important roles in the survival of all cell types and during proliferation and differentiation of neuroprogenitors (NPs), maturation and migration of neurons (Ns), as well as differentiation and maturation of astrocytes (Ass) and oligodendrocytes (Os). Black arrows indicate normal developmental processes. Red arrows and text represent inhibitory effects. Green arrows and text represent positive effects. Gray dotted arrow indicates *in vitro* dedifferentiation. iN, immature neuron; OP, oligodendrocyte progenitor; AsP, astrocyte progenitor; KAT, lysine acetyltransferase; KDAC, lysine deacetylase; HDAC, histone deacetylase; CBP, CREB-binding protein; VPA, valproic acid; TSA, trichostatin A; GCN5, general control of amino acid synthesis protein 5-like 2; TRRAP, transformation/transcription domain-associated protein; BRPF1, bromodomain and PHD finger-containing protein 1; MORF, MOZ-related factor; MOZ, monocytic leukemia zinc finger related protein; TIP60, Tat interacting protein 60 kDa; SIRT, sirtuin; ELP, elongator acetyltransferase complex subunit.

Clinical research as well as cellular and mouse models have revealed the critical role of a delicate control of the balance between lysine acetylation and deacetylation in brain development. Lysine acetylation and deacetylation are involved in all levels of brain development starting from NP survival and proliferation, cell fate decisions, neuronal maturation and migration, differentiation and maturation of astrocyte and oligodendrocyte lineages, and finally synapse formation and establishment of neuronal circuits (Figure 3). The complex, dynamic, and coordinated crosstalk between individual KAT and KDAC complexes in all these processes are not fully understood yet. Most mechanistic studies performed so far have largely focused on the role of histone acetylation in brain development, while neglecting the acetylation of other proteins. Recent studies have shown that acetylation of other proteins, such as tubulin or STAT3, also affects brain development [55], [56], [103]. Given the complexity of the regulatory networks, substrate redundancy, and lack of specificity of chemical inhibitors, it remains a challenge to determine the exact function of each KAT and KDAC enzyme, in association with each individual macromolecular KAT and KDAC complex, in regulating the acetylation of histones or other proteins. Perhaps with new large-scale approaches including large-scale genome editing, proteome, and acetylome analyses, as well as genome-wide transcriptome and epigenome analyses, new advances are anticipated in understanding the dynamic regulation, substrate specificity, and cellular functions of KATs and KDACs during brain development.

HDACi has been used to treat different human diseases, including cancer [164], cardiovascular diseases [164], inflammatory diseases [165], and a range of psychiatric disorders [21], [166]. Nevertheless, apart from the positive effects of HDAC inhibition on epigenetic brain disorders such as Rett syndrome and Fragile X-linked syndrome, a wealth of evidence shows that alterations of acetylation levels during brain development lead to dramatic negative effects and raising thus the question of whether chemical HDAC inhibitors are safe during developmental stages. Similarly, modulation of sirtuin activity is extensively studied for its positive effects on lifespan and aging [167]. However, the effects of sirtuins on crafting the brain development need to be carefully considered. Strikingly, a number of natural compounds found in commonly-consumed products are known to influence lysine acetylation through different mechanisms. Among others, HDAC inhibitors have been isolated from garlic and cruciferous vegetables, HDAC activators from apples, blueberries, and strawberries; and HAT inhibitors have been isolated from cashew nuts, green tea or curcuma [168], [169]. Moreover, lysine acetylation and deacetylation are greatly influenced by extracellular cues and metabolic states. However, the environmental regulation of KAT/KDAC activities and its effects on the establishment of the final brain structure remains elusive. The importance of protein acetylation and deacetylation during brain development has been largely overlooked. Future studies are a prerequisite to determine whether these processes exert a huge impact on the brain cytoarchitecture and function.

## Competing interests

The authors declare no competing financial interest.

## Figures and Tables

**Table 1:** KAT families and members

**Family**	**Name**	**Aliases**	**Location**	**Complexes**	**Other functions of the complex**
GNAT	HAT1	KAT1	Nucleus, cytoplasm	HAT-B	Chaperone activity
	GCN5	KAT2A, GCN5L2, HGCN5	Nucleus	TFTC, STAGA, ATAC	–
	PCAF	KAT2B	Nucleus	PCAF	–
	ELP3	KAT9	Nucleus, cytoplasm	Elongator	Transcriptional elongation in RNAPII complex
	CSRP2BP	KAT14	Nucleus	ATAC	–
	ATF-2	CREB2, CRE-BP1	Nucleus, cytoplasm, mitochondria	–	Transcription factor
	HAT4	–	Golgi	–	–
p300/CBP	CBP	KAT3A, CREBBP	Nucleus, cytoplasm	–	–
	P300	KAT3B, EP300	Nucleus, cytoplasm	–	–
MYST	TIP60	KAT5, HTATIP	Nucleus, cytoplasm	NuA4, TIP60, SWR1-like	Removal of H2A.Z/H2AFZ from the nucleosome (in SWR1-like complex)
	MOZ	MYST3, KAT6A, RUNXBP2, ZNF220	Nucleus	MOZ/MORF	–
	MORF	MYST4, KAT6B, KIAA0383, MOZ2	Nucleus	MOZ/MORF	–
	HBO1	MYST2, KAT7, HBOa	Nucleus	HBO1	–
	MOF	MYST1, KAT8, PP7073	Nucleus	MSL, NSL, MLL1/MLL	–
Others	TAF1	KAT4, BA2R, CCG1, CCGS, TAF2A	Nucleus	TFIID	Part of RNAPII complex
	TFIIIC	KAT12, GTF3C4	Nucleus	TFIIIC	RNAPIII transcription
	NCOA1	KAT13A, BHLHE74, SRC1	Nucleus	n.s.	Hormone–dependent transcriptional stimulation
	NCOA3	KAT13B, AIB1, BHLHE42, RAC3, TRAM1	Nucleus, cytoplasm	NCOA3/NCOA2/IKKA/IKKB/IKBKG/CBP	–
	NCOA2	KAT13C, BHLHE75, SRC2, TIF2	Nucleus	NCOA3/NCOA2/IKKA/IKKB/IKBKG/CBP	–
	CLOCK	KAT13D	Nucleus, cytoplasm	CLOCK, BHLHE8, KIAA0334	Regulation of circadian rhythm

*Note*: AIB1, amplified in breast cancer 1 protein; ATAC, Ada2a containing complex; ATF-2, activating transcription factor 2; BHLHE, class E basic helix-loop-helix protein; CREB, cAMP-responsive element-binding protein; CBP, CREB binding protein; CCG1, cell cycle G1 phase defect; CLOCK, circadian locomotor output cycles kaput protein 3; CSRP2BP, (cysteine and glycine rich protein 2) binding protein; ELP3, elongator acetyltransferase complex subunit 3; HAT, histone acetyltransferase; HBO1/HBOa, HAT binding to ORC1; IKBKG, inhibitor of kappa light polypeptide gene enhancer in B-cells kinase gamma; IKK, I-kappa-B kinase; GCN5/GCN5L2/HGCN5, (general control of amino-acid synthesis, yeast, homolog)-like 2; GNAT, gcn5-related *N*-acetyltransferase; GTF3C4, general transcription factor IIIC; HTATIP, HIV-1 Tat interacting protein, 60 kDa; KAT, lysine acetyltransferase; MLL1/MLL, myeloid/lymphoid or mixed-lineage leukemia 1; MOF, males absent on the first; MORF, MOZ-related factor; MOZ, monocytic leukemia zinc finger protein; MSL, male specific lethal; MYST, MOZ-Ybf2 (Sas3)-Sas2 and Tip60; NCOA, nuclear receptor coactivator; NSL, non-specific lethal; PCAF, p300/CBP-associated factor; RAC3, receptor-associated coactivator 3; RNAP, RNA polymerase; RUNXBP2, Runt-related transcription factor binding protein 2; SRC, steroid receptor coactivator; STAGA, SPT3-TAF9-GCN5 acetyltransferase complex; TAF, TATA-box binding protein associated factor; TFTC, TATA-binding protein-free TAF-II containing complex; TIF2, transcriptional intermediary factor 2; TRAM1, thyroid hormone receptor activator molecule 1; ZNF220, zinc finger protein 220; n.s., not specified; -, no information available.

**Table 2:** Classes of KDACs/HDACs

**Class**	**Name**	**Aliases**	**Location**	**Expression enrichment**	**Complexes**	**Other functions**
I	HDAC1	RPD3L1	Nucleus	Ubiquitous	Sin3, NuRD, CoREST, PRC2, others	–
	HDAC2	–	Nucleus, cytoplasm	Ubiquitous	HDAC1/HDAC2/RBBP4/RBBP7, RCOR/GFI/KDM1A/HDAC, SIN3, BHC, NuRD	–
	HDAC3	–	Nucleus, cytoplasm	Ubiquitous	N-CoR-SMRT	–
	HDAC8	HDACL1, CDA07	Nucleus, cytoplasm	Ubiquitous	–	–
IIA	HDAC4	KIAA0228	Nucleus, cytoplasm	Brain, skeleton growth plates	n.s.	–
	HDAC5	KIAA0600	Nucleus, cytoplasm	Heart, skeletal muscle, brain	n.s.	–
	HDAC7	HDAC7A	Nucleus, cytoplasm	Endothelial cells, thymocytes	n.s.	–
	HDAC9	HDAC7, HDAC7B, HDRP, KIAA074, MITR	Nucleus, cytoplasm	Heart, skeletal muscle, brain	n.s.	–
IIB	HDAC6	KIAA0901, JM21	Nucleus, cytoplasm	Heart, liver, kidney, placenta	n.s.	–
	HDAC10	–	Nucleus, cytoplasm	Liver, spleen, kidney	n.s.	–
III	SIRT1	SIR2L1	Nucleus, cytoplasm	–	–	–
	SIRT2	SIR2L, SIR2L2	Cytoplasm	–	–	–
	SIRT3	SIR2L3	Mitochondria	–	–	–
	SIRT5	SIR2L5	Mitochondria	–	–	Demalonylation, desuccinylation
	SIRT6	SIR2L6	Nucleus	–	–	ADP-ribosylation
	SIRT7	SIR2L7	Nucleolus	–	–	–
IV	HDAC11	–	Nucleus	Brain, heart, muscle, kidney, testis	n.s.	–

*Note*: GFI, growth factor independent; HDAC, histone deacetylase; HDRP, HDAC-related protein; KDM1A, lysine demethylase 1A; MITR, MEF-2 interacting transcription repressor; N-CoR, nuclear receptor co-repressor; NuRD, nucleosome remodeling deacetylase; PRC2, polycomb repressive complex 2; RBBP, retinoblastoma binding protein; REST, repressor element 1-silencing transcription factor; RCOR/CoREST, REST corepressor; RPD3L1, reduced potassium dependency yeast homolog-like 1; SIRT, sirtuin; SMRT, silencing mediator for retinoid and thyroid hormone receptors; n.s., not specified; -, no information available.

**Table 3:** Neurodevelopmental disorders associated with mutations or loss of function in KAT or KDAC complexes

**Gene**	**Molecular function**	**Human disease**		**Mouse model**		**Effects on neural development**
		**Disease**	**Refs.**	**Model**	**Refs.**	
*HAT1*	KAT enzyme	ASD	[142]	–	–	–
*p300*	KAT enzyme	Rubinstein Taiby syndrome; ASD	[119], [120], [121], [123], [124], [142], [170]	KO: early lethality; neural tube closure defects HI: memory deficits; behavioral impairment	[59], [62]	Regulation of NP differentiation
*CBP*	KAT enzyme	Rubinstein–Taiby syndrome; 16p13.3 duplication syndrome	[57], [117], [118], [125]	KO: early lethality; neural tube closure defects HI: memory deficits; behavioral impairment	[59], [60], [61], [63]	Regulation of NP differentiation
*PCAF*	KAT enzyme	ASD	[142]	KO: memory defects; abnormal hippocampal morphology	[64], [69], [70]	–
*GCN5*	KAT enzyme	ASD	[142]	KO: early lethality; apoptosis HM: neural tube closure defects; exencephaly *Nes*-Cre: microcephaly; decreased NSC mass	[65], [66], [67], [68]	Regulation of NP proliferation; essential for NP survival
*CRP2* B*P*	KAT enzyme	Human neurological disorders	[127]	–	–	–
*ELP3*	KAT enzyme	–	–	KD: cortical disorganization	[55]	Migration and maturation of neurons
*ELP1*	Component KAT complex	–	–	KD: cortical disorganization	[55]	Migration and maturation of neurons
*TIP60*	KAT enzyme	ASD	[142]	KO: early lethality (blastocyst)	[73]	Possible role in neurogenesis
*MORF*	KAT enzyme	Genitopatellar syndrome; Say-Barber-Biesecker syndrome; Noonan syndrome-like disorder	[78], [79], [128]	Gene-trap mutagenesis: neurodevelopmental defects	[76]	Required for NP proliferation
*MOZ*	KAT enzyme	Syndromic developmental delay with microcephaly and dysmorphic mutations	[129], [142]	KO: embryo or perinatal lethality	[80], [81]	Role in NP maintenance
*BRD1*	Component KAT complex	Schizophrenia; bipolar disorder	[130], [131], [132]	–	–	–
*Trrap*	Component KAT complex	–	–	KO: early embryo lethality *Nes*-Cre: severe brain atrophy	[74], [85]	Regulation of NP cell cycle and differentiation
*BRPF1*	Component KAT complex	–	–	KO: early embryo lethality *Emx1*-Cre: dentate gyros hypoplasia	[86]	NP maintenance
*CLOCK*	KAT enzyme	ASD	[142]	KO: brain phenotype not addressed	–	–
*HDAC1*	KDAC enzyme	ASD	[142]	*Nes*-Cre: no phenotype Double *Nes*-Cre *Hdac1*-*Hdac2*: neurodevelopmental defects	[88], [89]	–
*HDAC2*	KDAC enzyme	–	–	*Nes*-Cre: no phenotype. Double *Nes*-Cre *Hdac1-Hdac2*: neurodevelopmental defects	[88], [89]	Control of NP fate
*HDAC3*	KDAC enzyme	–	–	*Nes*-Cre: perinatal lethality; brain abnormalities	[90]	Regulation of cell fate determination
*HDAC8*	KDAC enzyme	Wilson-Turner X-linked mental retardation syndrome; Cornelia de Lange-like syndrome	[133], [134]	KO: perinatal lethality, skull instability	[92]	–
*HDAC4*	KDAC enzyme	ASD	[142], [171]	KO: perinatal lethality. *Thy1*-Cre/*Nes*-Cre: no phenotype	[94], [95]	–
*HDAC5*	KDAC enzyme	ASD	[142]	–	–	–
*HDAC7*	KDAC enzyme	ASD	[142]	–	–	–
*HDAC9*	KDAC enzyme	Schizophrenia; ASD	[137], [142]	KO: no brain phenotype described	[97]	–
*HDAC6*	KDAC enzyme	Chondrodysplasia with platyspondyly, distinctive brachydactyly, hydrocephaly and microphtalmia syndrome; ASD	[138], [139], [142], [172]	KO: brain phenotype not addressed	[96]	–
*HDAC10*	KDAC enzyme	ASD	[142]	–	–	–
*SIRT1*	KDAC enzyme	–	–	KO: defects in synaptic plasticity KD: defects in neurite outgrowth	[99], [100]	–
*SIRT6*	KDAC enzyme	–	–	KO: postnatal lethality; multiple abnormalities *Nes*-Cre: growth retardation	[104], [105]	–

*Note*: ASD, autism spectrum disorder; BRD1, bromodomain containing 1; BRPF1, bromodomain and PHD finger containing 1; CBP, CREB binding protein; CLOCK, circadian locomotor output cycles kaput protein 3; CRP2BP, cysteine rich protein 2 binding protein; ELP, elongator acetyltransferase complex subunit; GCN5, general control of amino acid synthesis protein 5-like 2; HDAC, histone deacetylase; HI, haploinsufficiency; HM; hypomorphic mutation; KAT, lysine acetyltransferase; KD, knockdown; KDAC, lysine deacetylase; KO, knockout; MORF, MOZ-related factor; MOZ, monocytic leukemia zinc finger protein; Nes, nestin; NP, neuroprogenitor; PCAF, p300/CBP-associated factor; TIP60, Tat interacting protein 60 kDa; TRRAP, transformation/transcription domain associated protein.
